# Processing methods for signal suppression of FTMS data

**DOI:** 10.1186/1477-5956-9-S1-S2

**Published:** 2011-10-14

**Authors:** Xuepo Ma, Jian Cui, Jianqiu Zhang

**Affiliations:** 1 Electrical and Computer Engineering Department, University of Texas at San Antonio, San Antonio, TX, USA

## Abstract

**Background:**

Fourier Transform Mass Spectrometry coupled with Liquid Chromatography(LC-FTMS) has been widely used in proteomics. Past investigation has revealed that there exists an intensity dependent random suppression in peptide elution profiles in LC-FTMS data. The suppression is homogenous for the same peptide but non-homogenous for different peptides. The correction of suppressed profiles and an estimation on the range of suppression are necessary for accurate and reliable quantification using FTMS data.

**Results:**

A software package, Gcorr, is presented. The software corrects peptide profiles that satisfy correction conditions, and it can predict fold change null distributions at different intensity levels. Subsequently, the significance P-values of measured fold changes can be estimated based on the predicted null distributions. We have used an 1:1 LC-FTMS label-free dataset pair collected based on the same sample to verify that our predicted null distributions conforms to that of the observed null distribution.

**Conclusions:**

This software is able to provide suppression correction for peptide profiles, suppression distribution analysis and peptide differential expression analysis in terms of its fold change significance. The software is freely available at http://compgenomics.utsa.edu/Suppression_Study.html.

## Background

Due to its capability in achieving high resolution and mass accuracy simultaneously, Fourier Transform Mass Spectrometry(FTMS) has gained popularity in quantitative analysis of biomolecules and biomaker discovery [1]. However, many researchers have found ion abundance accuracy of FTMS instrument problematic. Padley *et al*[2] make note of several sources of non-linearity in measurement. Schrader *et al*[3] also mention signal loss in large compound library experiment. Sterner *et al*[4] find that the signal of small proteins is suppressed by larger ones. Gordon *et al* find that due to ion interactions, the spectral signal intensities do not necessarily reflect true trapped-ion abundances [5]. Additional signal suppression phenomena due to the effects of measuring several peptides are brought up in [6].

In our previous work [7], we found that: 1)there exists signal suppression in Liquid Chromatrograhpy-FTMS (LC-FTMS) by investigating the isotope ratios between ^13^*C* and ^12^*C*; 2) the suppression is intensity dependent, the lower the intensity level, the severer the suppression; and 3)the suppression is non-homogenous for different peptide but homogenous for the same peptide. We developed a correction algorithm to correct peptide profiles with relative high intensity. For peptide profiles that cannot be corrected, we analyzed the range of suppression and it showed that 10% to 300% of measurement error could be resulted due to suppression.

Given such severe random suppression that affects a significant portion of peptides, the use of FTMS for biomarker discovery is questionable unless we can estimate the impact of random suppression. In this paper, we consider how the random suppression would affect fold change measurements of peptides between two label-free LC-FTMS samples or labeled LC-FTMS samples which is critical in differential analysis aiming for biomarkers discovery. Since many current biomarker discovery projects [8] employs LC-FTMS, our considered problem is critical.

Given a 1:1 label-free LC-FTMS dataset pair containing the same sample, because of experimental variation and random suppression, the measured peptide fold change is actually randomly distributed around 1:1, which is defined as the null distribution. We will need such null distributions to estimate the significance of measured fold changes in LC-FTMS experiments that compare two different samples. Note that since suppression characteristics change with intensity levels, null distributions also changes [7]. At a lower intensity level, due to significant random suppression, the null distribution generally has a large variance. At higher intensity levels, the random suppression effect is considerably less, and the null distribution is mainly caused by experimental variations. Generally, the null distribution at a given intensity level is not directly available in a regular differential LC-FTMS experiments, and we have to estimate them in order to provide significance P-values for all fold changes. Without estimating the appropriate null distributions, it would be hard to detect differentially expressed proteins reliably especially in the low intensity region. Currently no software provides such significance estimation or suppression correction. We develop a software, Gcorr, that performs correction/suppression characteristics analysis, and fold change significance estimation at different intensity levels.

## Method

**Figure 1:**
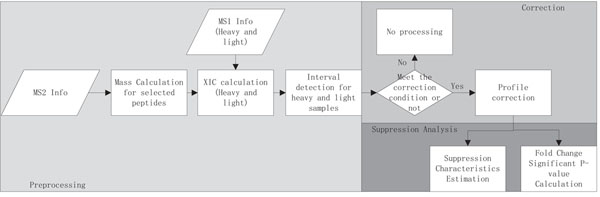
**Flow diagram of the software.** The flow diagram of the software.

**Figure 2:**
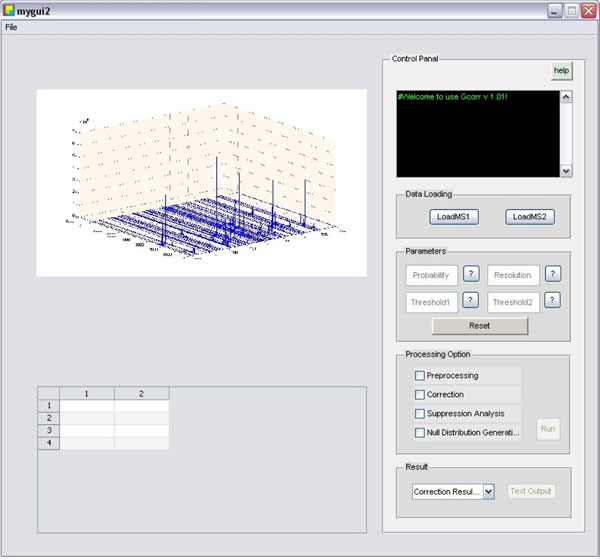
**The graphical user interface.** The graphical user interface.

The Gcorr software aims at correcting peptide profiles that satisfy correction conditions, providing suppression characteristic analysis, and estimating the significance levels of fold changes. The Gcorr software package consists of three parts: data preprocessing, profile correction and suppression analysis. In preprocessing, it performs mass calculation, extracted ion chromograph (XIC) calculation and peptide peak interval detection for each peptide of interests, for details see our previous work [7]. Then the elution profiles of peptides that meet correction conditions are corrected. Finally the overall suppression fold change characteristics is estimated based on corrected peptide profiles, and null distributions at different intensity levels are estimated for the calculation of significance P-values of measured fold changes. The flow diagram of Gcorr is shown in Figure [1]. We develop a graphical user interface which is shown in Figure [2].The tool box is implemented as a stand-alone MATLAB application, which is freely available at http://compgenomics.utsa.edu/Suppression_Study.html.

### Datasets used and data format

We use two replicate QC datasets and a UPS1 dataset for development and demonstration of Gcorr. The QC dataset is a quality control dataset generated on the organism LTQ_Orb_2. We downloaded the QC datasets DatasetQC_Shew_07_011pt0_b_04Feb07_Falcon_070202 and DatasetQC_Shew_07_011pt0_b_04Feb07_Falcon_070203 from http://omics.pnl.gov/browse/. For details of the data please see [9]. In the QC datasets, tandem peptide identification is performed at the same time of the LC/MS experiment and a peptide list annotated with sequence, charge state and elution time information is provided for each dataset. UPS1 is a Proteomics Standard Set (from *SIGMA – ALDRICH^TM^*), consisting of a mixture of 48 individual human source or human sequence recombinant proteins, each of which has been selected to limit heterogeneous post-translational modifications (PTMs). The total protein content in each vial is 10.6 mg. Each protein has been quantitated by amino acid analysis (AAA) prior to formulation. All these datasets are collected on LC-FTMS.

Both LC-MS(MS1) data and LC-MS/MS (MS2) data are required to run the software. Currently, the software support MS1 data in mzxml format and MS2 data in excel format. Many instruments support the mzxml format, and many tools are available to convert .raw to .mzxml files, . We require that MS2 files in excel format must be the output of Trans-Proteomic Pipeline(TPP) [10], the MS2 data file after TPP contains a list of peptides that have been identified, and need to be quantified. Most importantly, the MS2 peptide list is annotated with PeptideProphet [11] scores which allows us to pick existing peptides for suppression characteristic analysis. More detail of the data format is provided in the supplemental material. After the MS1 and MS2 information are loaded, the mass, XIC and peak interval of identified peptide are calculated. As the preprocessing could be very time consuming(depend on the size of the input data and the number of peptide of interest), the output of mass, XIC and peak interval values can be exported as MATLAB .mat files and stored for future uses. The output of the software is a peptide list in text format. The result file include these information: the peptide sequence, the abundance before correction, whether the peptide is correctable, abundance after correction and the p-value of fold change. The Gcorr software takes these input files: 1) Two label free LC-FTMS files that contains samples of two classes to be compared; 2) A list of peptides to be differentially analyzed; and 3) a null distribution file that describes experimental variations. Given these inputs, Gcorr outputs: 1) Corrected peptide profiles and their corrected fold changes if they can be corrected. 2) Fold change significance P-values for peptides that can not be corrected. The Gcorr software can be easily extended to process labeled LC-FTMS datasets.

### Correction function estimation and correction conditions

In this section, we briefly describe the correction function in Gcorr. In [7], we developed an peptide profile correction algorithm based on iterative conditional mode (ICM) algorithm. Given a peptide, we denote its observed elution profiles as **y**_1_ = [*y*_1_(*t*_1_), *y*_1_(*t*_2_), ⋯] for the higher peptide profile, and **y**_2_ = [*y*_2_(*t*_1_), *y*_2_(*t*_2_), ⋯] for the lower profile of ^12^*C* and ^13^*C* respectively, where *t*_1_, *t*_2_, ⋯ are sampling time of the elution profiles. Define **x**_1_ and **x**_2_ as the true profiles of the peptide. **x**_1_ and **y**_1_ are related as **x**_1_ = *f*(**y**_1_), where *f*(*·*) is the correction function. We define the inverse function of *f*(*·*) as the distortion function *g*(*·*)*.* We verified in our previous work, that the distortion of different isotopes is the same for the same peptide, and we have **x**_1_ = *f*(**y**_1_) and **x**_2_ = *f*(**y**_2_). Let **T** = **x**_1_ + **x**_2_ represents the total ion count of the peptide at ^12^*C* and ^13^*C*. The basic idea is: 1.Set an initial value for the total ion count **T** = **y**_1_ + **y**_2_. 2. Based on the total ion count, the isotope ratio *r* and **y**_2_, calculate the most probable correction function. 3. Correct the elution profiles **y**_1_ and **y**_2_ using currently estimated correction functions, and the values of **x**_1_ and **x**_2_ are updated. 4. Estimate a new correction function with the updated **x**_2_, the isotope ratio and the total ion count **T**. 5. Repeat step 4, and 5 until the convergence condition is met. We can obtain the correction function and the corrected peptide profiles after the application of the algorithm. For detail of the algorithm please refer to [7]. However, the algorithm can only be used to correct peptide profiles that satisfy these correction conditions: 1. The isotope ratios are not close to zero or one (**x**_2_*/***x**_1_ = 0.2 to 0.8 for example), since in these cases, the suppression is not detectable by comparing **y**_1_ and **y**_2_; 2.The maximum intensity of **y_2_** need to be greater than the distortion free range lower limit (10^6^) , as the function can only be estimated on the range that **y_2_** spans, and if max(**y_2_**) does not reach the lower limit, then the correction function cannot be found for the full range that needs correction.

### Suppression characteristics study

The correction algorithm corrects profiles that meet the correction condition almost perfectly. However, only part of peptide profiles with **y_2_** reaching certain threshold can be corrected. In general, such threshold is very high, and the intensity of most peptides (70% - 90%)are lower than the threshold [7]. We want to understand the characteristics of those uncorrectable peptides. We consider the correction functions that has been found as random samples of all possible correction functions, based on which, we can estimate the overall suppression characteristics. We want to know how the peptide profiles are suppressed at different intensity levels. To estimate suppression characteristics, we apply random correction functions to typical peptide profiles at lower intensities to get an estimation of the range of suppressions at different intensity levels.

**Figure 3:**
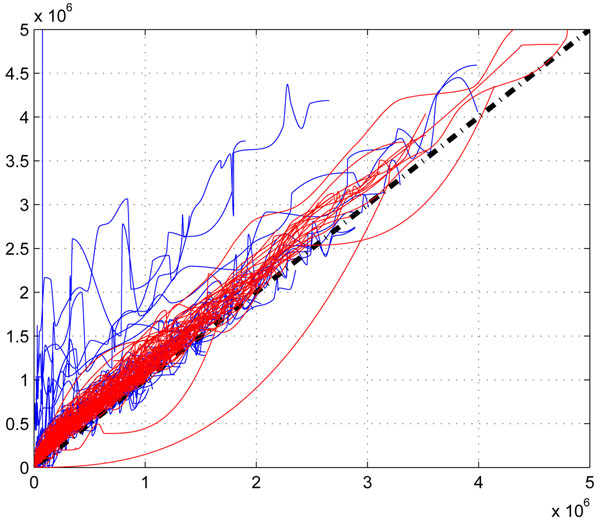
**The comparison of correction functions of UPS1 data and QC data.** The comparison of correction functions of UPS1 data and QC data. The red lines are the correction functions of UPS1 data, and the blue lines are the correction functions of QC data.

**Figure 4:**
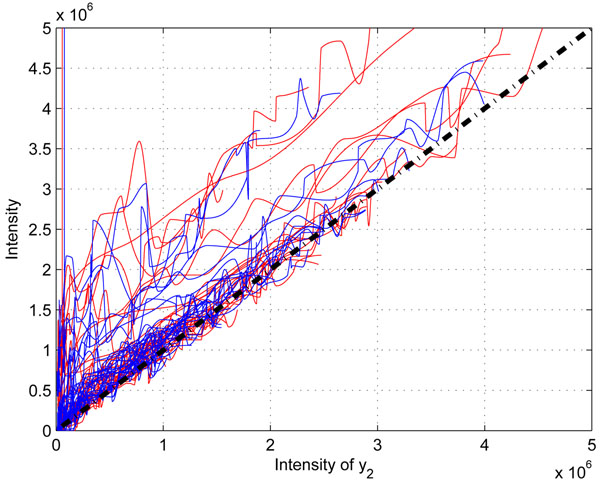
**The comparison of correction functions of two QC replicate data(QC02 and QC03).** The comparison of correction functions of two QC replicate data(QC02 and QC03). The red lines are the correction functions of QC02 data, and the blue lines are the correction functions of QC03 data.

Our investigation reveals that the suppression is different in different labs. In Figure [3], we plot the correction functions of two different datasets (UPS1 and QC) collected from two labs, we can see that there exits an obvious difference of the two set of correction functions. However, for replicate QC data, their correction functions are is similar as shown in Figure [4]. This shows that in the same lab with identical experimental conditions, we can assume similar statistical characteristics in correction functions.

### Fold change variation analysis

Once we obtain samples of correction functions for each LC-MS dataset, we can further examine the fold change variation of peptides from different samples in differential analysis using LC-FTMS. The variations in measured fold change are caused by experimental and instrumental variations. These variations need to be considered carefully in biomarker discovery, as the variation are intensity dependant. Gcorr provides a tool for evaluating whether fold changes of peptides are significant.

#### Experimental and instrumental variation

There are two kind of variations in the FTMS data. One is experimental variation which is caused by sample preparations and other experimental steps. The other is instrumental variation which is the result of random suppression.

Experimental variations can be obtained by inspecting the measured ratios between identical peptides in two datasets that are supposed to contain the same amount of such peptides. The most convenient way to get such a null distribution is to inspect a 1:1 label-free LC-FTMS dataset pair collected based on the same sample. We have found that the suppression is intensity dependent in our previous work, and when the intensity of a peptide profile is greater than a certain threshold, the observed profile is suppression free. The variation in measured fold changes of suppression free peptide profiles is only determined by the experimental process, and we can consider the fold change distribution of suppression free peptide profiles as the experimental null distribution. For the profiles that is lower than the threshold, they suffers from both experimental and instrumental variation. As the suppression is intensity dependent, the resulted null distributions are different at different intensity levels and they can not be considered as experimental distributions. Several runs of 1:1 data can be collected to further confirm the experimental variation in a particular set of experimental conditions. If 1:1 dataset are not available, identical amount of peptide standard can be spiked in two samples for experimental null distribution estimation.

**Figure 5:**
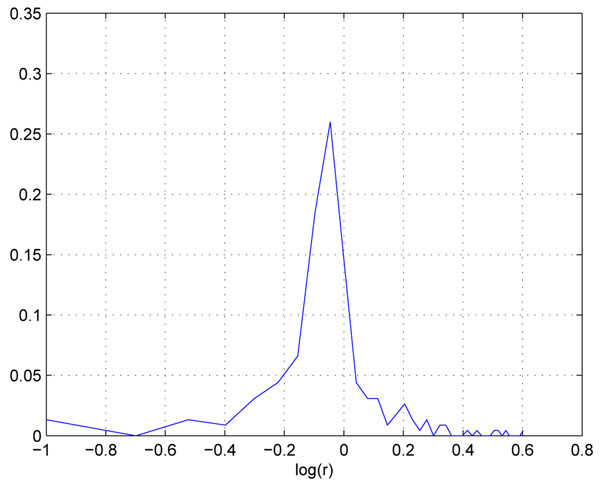
**The foldchange distribution of suppression free peptide profiles.** The foldchange distribution of suppression free peptide profiles.

**Figure 6:**
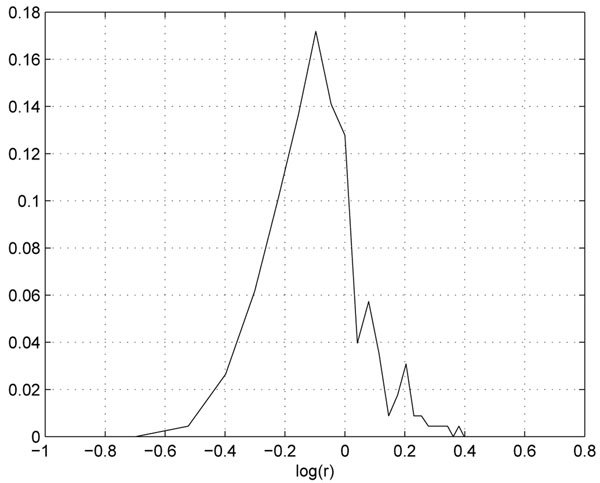
**The foldchange distribution of peptide profiles suffering from both experimental variation and instrumental variation.** The foldchange distribution of peptide profiles suffering from both experimental variation and instrumental variation.

For example, in the two replicate QC datasets, the sample ratio is 1:1, and we found that profiles are generally distortion free if the intensity is greater than certain threshold(10^6^ in this case). We can use the higher portion of the profiles to estimate the effect of the experimental variation. Figure[5] is the log abundance ratio distribution of the distortion free peptide profiles from the two replicate data. We can see the distribution is much narrower than the overall distribution(centered at -0.05). The variation of profiles that are lower than 10^6^ can be attributed to from both experimental and instrumental variations. For example, assuming that due to experimental variation, the abundance ratio of a particular peptide is 0.9. Subsequently in FTMS, the profile from the less abundant sample will be suppressed more than that in the more abundant sample, and as a result, the abundance ratio may be measured at 0.85. Figure [6] shows the log abundance ratio distribution of the suppressed peptide profiles. We can see that the ratios are further lowered(centered at -0.1) and the variance has increased.

From these observations we hypothesize that the overall variation if measured fold changes is caused by experimental variations and instrumental random suppression. While the experimental variations are not intensity dependant, the instrument variation is. Under this hypothesis, if given experimental variations of an typical differential LC-FTMS experiments, we can get a null distribution of fold changes at any given intensity level with the collection of estimated correction/distortion functions. Then base on null distributions at different intensity levels, we can provide a significance p-value for any measured fold change between two samples. Note that generally the null distribution is not directly available in a typical differential LC-FTMS experiment due to different sample contents.

To test the hypothesis, here we show that for a 1:1 label-free dataset pair, we can predict a null distributions that conforms to the observed null distribution at different intensity levels after we transform an experimental null distribution by estimated correction/distortion functions. Note that with a 1:1 dataset pair collected based on the same sample, it is possible to get the observed null distribution on fold changes at different intensity levels. However in most cases, such distributions are not available.

**Figure 7:**
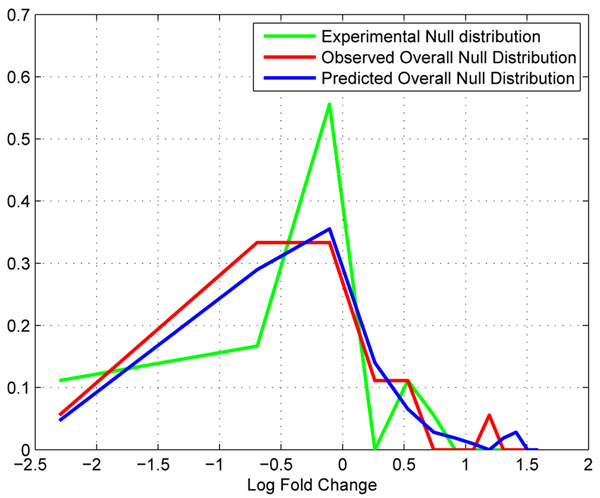
**Comparison of the observed null distribution and the predicted null distribution.** Comparison of the observed null distribution and the predicted null distribution.

In replicate QC datasets, 18 peptide profile pairs satisfy the correction conditions, and their correction functions are obtained using the ICM algorithm [7]. We want to (1) extract experimental null distributions form unsuppressed parts of the profiles that are greater than a threshold 10^6^. (2) We want to see how the experimental null distribution would be further spread due to instrumental suppression at a given intensity level. To accomplish this, we first sample randomly corrected peptide profiles **y***_p_* and scale them to desired intensity range. Then we take a sample from the experimental null distribution, if the sampled fold change is *a^j^* (*j* indicates *j*th sample from the experimental null distribution), then we pretend that **y***_p_* as the sampled profile in one LC-MS dataset, and *a^j^* · **y***_p_* as the peptide profile from the other dataset. We then apply all distortion functions derived from one LC/MS datasets as, and . We then record  as the predicted ratios after random suppression, where *g_i_*(*·*)s are distortion functions, *i* ∈ (1, 2,*…*, *N*) where *N* is the total number of sample correction/suppression functions. We repeat this process for different samples from the experimental null distribution. The set of *r^ij^* will form a predicted null distribution of fold changes at the considered intensity level. Subsequently, based on the 1:1 dataset pair, we found 18 peptide profiles that are within the same intensity range as that of the predicted null distribution. We consider the fold change distribution of these peptides as the observed one. Figure [7] illustrates the predicted null distribution and the observed one, we can see that the two distributions are similar to each other. We use the Kolmogorov-Smirnov Test to determine if the two distribution differs [12]. The null hypothesis is that the two distributions are the same. Calculation result fail to reject the null distribution with a p-value 0.17. This result show that our hypothesis is valid and we can use predicted null distributions for fold change significance analysis in the next step.

#### Significance Estimation

As the experimental null distribution and the overall null distribution are base on 1:1 label free samples, it is expected that a lab will first conduct a 1:1 sample run to get an estimation of experimental variation using the same set of equipments.

Subsequently at a regular differential experiment, the Gcorr software would be applied to find correction/distortion functions. Subsequently, given a fold change measurement between two peptides, we will first determine its intensity range and estimate its overall null distribution based on the set of estimated correction/distortion functions. Once we have obtain the null distribution estimation, then the fold change’s significance score will be calculated based on the null distributions.

## Test on two label-free QC datasets

**Figure 8:**
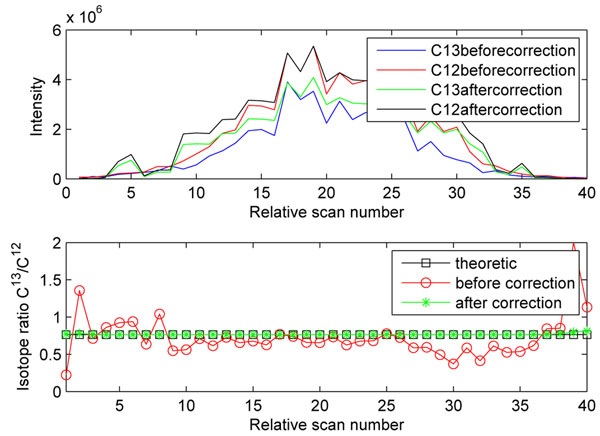
**Correction result of one peptide.** Correction result of one peptide.

**Figure 9:**
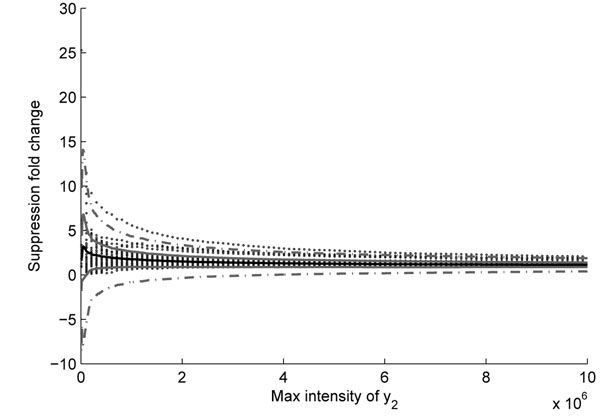
**Fold change statistics of QC data.** The black solid line is the mean value of suppression at different level; the gray solid line is mean value ± standard deviation; the gray dash line is mean value ±3× standard deviation.

The QC dataset has two replicates. We load the MS1 and MS2 data from both QC datas, then the mass, XIC and peak interval are calculated. With the preprocessed data, the peptides that meet the correction conditions are corrected. Figure [8] is the correction result of one peptide. Using peptide profiles that meet the correction condition, the suppression characteristics can be estimated, Figure [9] is the fold change statistics. After these steps we performs experimental null distribution estimation, and for each measured fold change we estimate its overall null distribution based on the intensity of the taller profile. Then Gcorr estimate the significance p-value for all the fold changes. The output files of this experiment are posted on the website http://compgenomics.utsa.edu/Suppression_Study.html.

## Conclusions

We develop a software for correcting the signal suppression in FTMS data based on the interactive correction mode algorithm. The software have been tested with replicate QC data, part of peptides satisfying the correction condition can be corrected perfectly. With the corrected peptide profiles, the overall null distribution is estimated and compared to the theoretical prediction at a lower intensity level. Based on such null distributions, the significance P-value of fold changes in a typical LC-FTMS differential analysis experiment can be calculated.

## Competing interests

The author(s) declare that they have no competing interests.

## Authors contributions

XM implemented the software, performed simulations and wrote the first draft of the paper. JC assisted the development of the software. JZ conceive the concept of the software, advised the development of the software and revised the manuscript.
